# Genetics of Ribosomal Proteins: “Curiouser and Curiouser”

**DOI:** 10.1371/journal.pgen.1003300

**Published:** 2013-01-31

**Authors:** Tamara Terzian, Neil Box

**Affiliations:** University of Colorado Denver, Dermatology Department and Center for Regenerative and Medicine and Stem Cell Biology, Aurora, Colorado, United States of America; Stanford University School of Medicine, United States of America

## The Mystery of *Minutes*

Lewis Carroll wrote about Alice, but he might just as well have been referring to Calvin Bridges. As a student in T.H. Morgan's lab, Bridges described some of the earliest *Drosophila* mutations, including so-called *Minutes*, in which heterozygotes exhibited small body size and developmental abnormalities in tissues undergoing rapid cell division, and homozygotes were lethal [1]. At the time, it was curious how dozens of different loci could yield the same phenotype, and even curiouser how flies multiply heterozygous at different *Minute* loci were no more severely affected than a single *Minute* mutant. This mystery—how dozens of genes could encode similar but separate proliferative functions in all cells—was solved more than 50 years later with the realization that mutations of ribosomal protein genes occur in almost all *Minute* loci [2].

In this issue of *PLOS Genetics*, Watkins-Chow et al. [1] add to a more recent curiosity: even though ribosomes (and ribosomal protein [RP] genes) have remained nearly identical across more than a billion years of evolution, mutations of RP genes in mice and in humans give rise to a surprising diversity of phenotypes. This work adds a new piece to a very old puzzle, and suggests the possibility that RP genes do more than just contribute to ribosomes.

## RP Mutations in Mammals: More than *Minutes*

To date, 11 different RP mutant mice have now been reported. These mice carry deletions, missense, or splicing mutations that have arisen spontaneously, from *N-Ethyl-N-Nitrosurea (ENU)* mutagenesis screens, or from targeted gene deletion [1]–[11] (Table 1). Overall, RP mutant mice exhibit an unexpected array and diversity of phenotypes. For example, the spontaneous mutant *Belly spot and tail* is caused by a splicing abnormality in *Rpl24* [6]; heterozygous mutants (*Rpl24^Bst/+^*) are small with white hind feet, a midline belly spot (*Bst*), abnormal retinal development, and skeletal abnormalities that include a curly tail [4]. Heterozygosity for a targeted mutation of *Rps6* causes embryonic lethality [12], while heterozygosity for targeted mutations of *Rpl22* or of *Rpl29* have no effect. (Homozygosity for targeted mutations of *Rpl22* and of *Rpl29* causes a T-cell–specific developmental defect [6] and generalized reduced growth [8], respectively.) Mice heterozygous for mutations in *Rps19*, *Rps20*, or *Rpl27a* exhibit epidermal hyperpigmentation, anemia, and reduced body size while the *Rpl27a* heterozygotes also exhibit cerebellar ataxia. *Rps19*-null mice are embryonic lethal prior to implantation [10]. *CD74-Nid67^+/−^* mice with a heterozygous deletion of eight genes including *Rps14* developed macrocytic anemia and other hematopoietic defects [11]. Finally, heterozygous *Rpl38* mutants had abnormalities of skeletal patterning [9].

**Table 1:** Mouse Models of Ribosomal Proteins.

Gene	Protein Synthesis Defect	Phenotypes	p53-Dependent	Refs
***Rpl24***	Decreased global protein synthesis	*Bst/+:* Small body size, bell spot and abnormal skeletal and eye morphology*Bst/Bst*: Embryonic lethal	YesN/A	[2] [4]
***Rps6***	N/A	*+/−:* Embryonic lethal	Partial	[12]
***Rpl22***	N/A	+/−: No phenotype*−/−:* Viable, defect in alpha-beta T-cells	Yes	[6]
***Rpl29***	Decreased global protein synthesis	*+/−:* No phenotype*−/−:* Viable, mild growth retardation	Yes	[8]
***Rps19***	N/A	*Dsk3/+:* Small body size, belly spot, anemia and epidermal hypermelanosis*Dsk3*/*Dsk3:* Embryonic lethal*+/−:* No phenotype*−/−:* Embryonic lethal	YesN/AN/AN/A	[7] [28] [10] [10]
***Rps20***	N/A	*Dsk4/+:* Small body size, belly spot, anemia and epidermal hypermelanosis*Dsk4*/*Dsk4*: Embryonic lethal	YesN/A	[7] [28]
***Rps14*** 1		*CD74-Nid67^+/−^* Macrocytic anemia*CD74-Nid67^−/−^* N/A	Yes	[11]
***Rpl27a***	N/A	*SFA*/*+*: Small body size, pancytopenia, epidermal hypermelanosis, cerebellar ataxia*SFA/SFA*: Embryonic lethal	YesNo	[5]
***Rpl38*** 2	Translational control of *Hox* mRNAs	*Ts/+* : Skeletal patterning abnormalities*Ts/Ts*: Embryonic lethal	NoN/A	[9]
***Rps7***	18S rRNA pre-processing	*Zma/+:* Small body size, belly spot, skeletal, eye and neuro-anatomical defects. 74% Viability (BALB/cJ×C57BL/6J), 100% embryonic lethal on N4 C57BL/6J.*Zma/Zma*: Embryonic lethal*Mtu/+:* Small body size, belly spot, skeletal and neuro-anatomical abnormalities. 26% Viability on C3H/HeJ background.*Mtu/Mtu*: Embryonic lethal	YesN/AN/AN/A	[1]

1 *Rps14* is one of eight genes deleted in the *CD74-Nid67^+/−^* mouse. It is the major candidate gene.

2 *Rpl38* is also mutated in the *Tail-short Shionogi* (*Tss*) and *Rabo torcido* (*Rbt*) mice.

*+/−:* heterozygous gene deletion; *−/−:* homozygous gene deletion; rRNA: ribosomal RNA. N4:4^th^ generation cross.

Mouse RP mutations: *Bst: Belly spot; Dsk*: *Dark skin*; *SFA*: *Sooty Foot Ataxia*; *Ts*: *Tail short*; *Mtu*: *Montu*; *Zma: Zuma*.

Watkins-Chow et al. [1] present two different missense alleles of *Rps7*, *Montu* (*Mtu*) and *Zuma* (*Zma*), that were generated from an *ENU* mutagenesis screen. Most *Rps7^Mtu/+^* mice die *in utero* (74% on a C3H/HeJ background), but the survivors show pleiotropic phenotypes including reduced body size, abnormal skeletal morphology, and mid-ventral white spotting. This phenotype cluster is reproduced in *Rps7^Zma/+^* mice and is similar to that in *Rpl24* mutant mice [1]. These observations support the existence of distinctive spatial and temporal characteristics for RPs [13]: Rps7, Rps19, Rps20, and Rpl24 may be necessary for melanocyte development [1], [2], [4], [7], while Rps6, Rps19, Rps20, and Rpl27a are important for keratinocytes [3], [7]. Additionally, Rps7, Rpl24, and Rpl38 are crucial for skeletal and retinal development [1], [2], [9] and Rps14, Rps19, Rps20, and Rpl27a are necessary for hematopoeisis [3], [7], [11]. RP genotype–phenotype correlations are also found in humans where mutations cause diseases with similarly complex clinical manifestations such as Diamond Blackfan anemia (DBA). DBA is characterized by diverse abnormalities including anemia, congenital craniofacial malformations, and defects in kidney development [14].

## The Role of p53 in RP Haploinsufficiency Phenotypes

While disturbances in ribosome biogenesis have been linked to many human diseases [15], there is also increasing evidence that RP mutations may be associated with cancer susceptibility [15]–[17]. In keeping with these observations, a sensitive connection between RPs and the tumor suppressor p53 has been identified (reviewed in [18]). p53 is a major cellular stress sensor that is best known for its ability to induce apoptosis, cell cycle arrest, and senescence in response to a variety of insults including DNA damage, oncogene activation, hypoxia, and more recently, ribosomal stress [19]. Thus, the p53 pathway provides a surveillance mechanism for the preservation of genomic and ribosomal integrity. Perturbation of ribosomal integrity induces the release of several RPs from the nucleolus that interact with and suppress the activity of the main negative regulator of p53, Mdm2, leading to the stabilization and activation of p53 [20]–[25]. This is observed in a number of RP mutant mice that exhibit cell cycle arrest and apoptosis in the affected cell types and in which the resulting pathologies are ameliorated or suppressed by *p53* deletion (Table 1). In *Rps19^Dsk3/+^*, *Rps20^Dsk4/+^*, *Rpl24^Bst/+^*, *Rpl27a^Sfa/+^*, and *Rps7^Zma/+^* mice, the removal of one *p53* copy is sufficient to alleviate all phenotypic abnormalities [1]–[3], [7]. In the case of *Rps6^+/−^* mice, fetal mortality is delayed by only a couple of days in the absence of *p53* [12]. Amazingly, the embryonic lethality of *Rps7^Zma/+^* mice was completely suppressed by the loss of one *p53* allele, and *Rps7^Zma/+^*:*p53^+/−^* mice are for the most part identical to their wild-type littermates [1]. Taken together, these experiments establish p53 as a true sensor of nucleolar stress and highlight the extraribosomal activity of RPs as modulators of the Mdm2–p53 pathway.

## RP Functions in Mutant Mice

Mutant RP phenotypes in mice appear to be the result of three distinct mechanisms: 1) global suppression of protein synthesis; 2) specific suppression of protein synthesis; and 3) extra-ribosomal functions. Diminished global protein synthesis was identified in *Rpl24^Bst/+^* (∼30% reduced) and *Rpl29^−/−^* mouse neural tube and somites (∼45% reduced), although no concurrent phenotype was described [9]. In *Rps7^Mtu/+^* mice, a novel defect in 18S rRNA preprocessing was identified in brain and liver, without a reduction in protein synthesis. We speculate that an accompanying reduction in protein synthesis could explain the homozygous embryonic lethality in these mice. On the other hand, the abnormal homeotic transformations observed in *Rpl38^Ts/+^* mice were attributed to the unique role of Rpl38 in translation of specific *Hox* mRNAs rather than to a global effect [9]. RPL38 appeared to facilitate 80S complex formation during the earliest steps of translation initiation on selective Hox mRNAs. In addition to these global and gene-specific translational defects, extra-ribosomal functions of numerous RPs have been described in prokaryotes and lower eukaryotes such as yeast and flies, and in cultured human cells [13], [26], [27]. This expanding list of functions includes cellular apoptosis, transcription and mRNA processing, DNA repair, development, and tumorigenesis. To date, the ability of RPs to activate p53 is the only described extra-ribosomal function in mice. In *Rps7^Mtu/+^* mice, impaired rRNA preprocessing and p53 activation occur simultaneously, illustrating the complex roles of RPs in mammalian tissues. With this in mind, mouse RP phenotypes (heterozygous and homozygous) need to be analyzed on a *p53*-null as well as on a p53 wild-type genetic background. Since so little is known about how changes in ribosomal protein levels may impact cellular function *in vivo*, a full repertoire of mouse RP models and improved characterization are sorely needed.

Due to the essential nature of RPs, it is apparent that even small perturbations to their functions can result in an increasing array of diseases, and it is clear now more than ever that the mystery of the underlying mechanistic basis for RP mutant phenotypes needs to be solved before state-of-the art translational approaches can bring effective treatments. Moreover, additional mouse models for RP disorders will provide an important preclinical resource for developing new treatments of ribosomopathies.
